# Balanced Translocation Disrupting *JAG1* Identified by Optical Genomic Mapping in Suspected Alagille Syndrome

**DOI:** 10.1155/2023/5396281

**Published:** 2023-06-08

**Authors:** Yi-Qiong Zhang, Peng-Fei Gao, Jing-Min Yang, Jing Zhang, Yu-Lan Lu, Jian-She Wang

**Affiliations:** ^1^The Center for Pediatric Liver Diseases, Children's Hospital of Fudan University, National Children's Medical Center, Shanghai 201102, China; ^2^State Key Laboratory of Genetic Engineering, School of Life Sciences, Fudan University, Shanghai, China; ^3^Shanghai WeHealth Biomedical Technology Co., Ltd., Shanghai, China; ^4^Key Laboratory of Birth Defects and Reproductive Health of National Health and Family Planning Commission (Chongqing Key Laboratory of Birth Defects and Reproductive Health, Chongqing Population and Family Planning, Science and Technology Research Institute), Chongqing 400020, China; ^5^Center for Molecular Medicine, Shanghai Key Lab of Birth Defects, Pediatrics Research Institute, Children's Hospital of Fudan University, Shanghai, China; ^6^Shanghai Key Laboratory of Birth Defect, Shanghai, China

## Abstract

We report the clinical and genetic features of a Han Chinese boy who presented with disease suspect for Alagille syndrome (ALGS). Multiple genetic analyses (panel sequencing, multiplex-ligation-dependent probe amplification, and whole genome sequencing) failed to uncover a causative variant. Optical genomic mapping detected a reciprocal translocation between chromosomes 4 and 20, interrupting *JAG1*. Long-range polymerase chain reaction and targeted sequencing identified the exact breakpoints. Sanger sequencing and reanalysis of genome sequencing raw data further confirmed the result. This translocation is expected to generate aberrant *JAG1* transcripts that lead to complete loss of *JAG1* expression. This is the first t(4;20)(q22.1;p12.2) balanced translocation detected by optical genomic mapping and characterized at base-pair resolution in ALGS. Our approach permitted precise diagnosis and genetic counseling.

## 1. Introduction

Alagille syndrome (ALGS; OMIM# 118450) is a multisystem autosomal dominant disorder with a wide variety of clinical manifestations, including hepatic, cardiac, skeletal, ophthalmologic, and facial abnormalities [1]. Clinical features differ even within the same family. The genetic etiology of ALGS is variation in *JAG1* or *NOTCH2*, which respectively encode a fundamental ligand and receptor in the Notch signaling pathway. Pathogenic variants in *JAG1* account for ~95% of disease burden in ALGS, while those in *NOTCH2* account for ~2.5%. Gene panels, multiplex-ligation-dependent probe amplification (MLPA), and whole exome/genome sequencing (WES/WGS) have been used to discover causative variants. Combinations of multiple methods have greatly improved diagnosis rates [2]. However, unresolved cases remain [3].

We identified a *de novo JAG1*-*FAM13A* gene fusion, with balanced reciprocal translocation, by optical genomic mapping (OGM) and confirmed it by targeted sequencing in an ALGS patient in whom initial genetic tests (panel sequencing, MLPA, and WGS) failed to detect a causative variant in *JAG1* or *NOTCH2*. This suggests that OGM has a role in detecting complex structural variants (SVs) in ALGS-related genes.

## 2. Patient and Methods

### 2.1. Clinical Data

The patient was a 3-year-old Han Chinese boy, born vaginally at 40 weeks' gestation and weighing 2330 g. Jaundice persisted from birth, with elevated values for serum direct bilirubin concentrations and aminotransferase and gamma-glutamyl transferase activities. He was referred to our center aged 72 days for investigation. Physical examination revealed short stature (both weight and height <3^rd^ percentile), facial dysmorphism (prominent forehead, deep-set eyes, and small pointed chin), yellow staining of skin and sclera, hepatomegaly, and splenomegaly. No posterior embryotoxon was found on ophthalmologic examination. Auscultation revealed a midsystolic murmur, grade 2/6, over the pulmonary valve area. Biomarker assays confirmed cholestasis with hypercholesterolemia (Table 1). Abnormal urinary microprotein profiles suggested mild renal tubule injury (Table 1). Infection with *Toxoplasma gondii*, rubella virus, cytomegalovirus, herpes simplex virus, and hepatitis viruses was excluded by serological screening. An anteroposterior radiogram of the spine showed a vertebral cleft (Figure S1). Color Doppler echocardiography indicated left pulmonary artery stenosis and persistent left superior vena cava. Clinical, biochemical, and imaging-study findings strongly suggested ALGS [4].

### 2.2. Panel Sequencing and MLPA

Genomic DNA (gDNA) from peripheral blood of the child was isolated using QIAamp DNA Blood Mini Kit (51106; Qiagen, Hilden, Germany). Panel sequencing was done as described [5], with gDNA fragments enriched using the ClearSeq Inherited Disease panel kit (Agilent, Santa Clara, CA). DNA libraries were sequenced on a HiSeq 2500 sequencer (Illumina, San Diego, CA) according to manufacturer's instructions. 90 bp paired-end sequencing reads were obtained, with at least 100-fold average sequencing depth. MLPA analysis was performed as described [6], using SALSA MLPA Probemix P184 JAG1 kit (MRC-Holland, Amsterdam, Netherlands) according to manufacturer's instructions.

### 2.3. Short-Read Whole Genome Sequencing (S-WGS)

S-WGS was executed as described [7], with gDNA isolated as for panel sequencing. Genome libraries were constructed using the TruSeq Nano DNA HT Sample Prep Kit (FC-121-4003; Illumina) and sequenced on the Illumina NovaSeq 6000 platform (Illumina) to generate 150 bp paired-end reads. FASTQ files were analyzed on the DRAGEN Bio-IT Platform (version 3.2.8, Illumina) to generate the variant call for single-nucleotide variants (SNVs), insertion-deletion variants (InDels), copy number variants (CNVs), and SVs. Reads were mapped to reference sequence GRCh38/hg38 with an average sequenced coverage of 25.48 and mapping rate of 99.46%.

### 2.4. Optical Genomic Mapping

High-molecular-weight patient DNA was extracted from fresh whole blood using Prep Blood and Cell Culture DNA Isolation Kit (30033; Bionano Genomics, San Diego, CA). OGM and annotation of gDNA were performed at WeHealth Biomedical Technology (Shanghai, China). DNA labeling was processed with Prep DLS DNA Labeling kit (80005; Bionano Genomics) according to the kit protocol. In brief, DNA was labeled with DLGreen fluorophores (20351; Bionano Genomics) using direct labeling enzyme 1 (DLE-1; 20351; Bionano Genomics) at 37°C for 2 h, digested with proteinase K (158920; Qiagen) at 50°C for 30 min, and cleaned up with 1x DLE-1 buffer (20350; Bionano Genomics). Subsequently, the DNA backbone was subjected to DNA staining (20356; Bionano Genomics), 5x dithiothreitol (20354; Bionano Genomics), and 4x flow buffer (20353; Bionano Genomics) for 1 h and mechanically homogenized (WH-986; Kylin-Bell, Haimen, China) overnight at 4°C. Labeled DNA was loaded on a Saphyr chip (Bionano Genomics) and processed using a Saphyr instrument (Bionano Genomics). Total depth was 100x. Quality control criteria included (1) half of DNA molecules ≥ 150 kbp, (2) mapping rate ≥ 70%, and (3) effective coverage > 80x. *De novo* genome map assembly was performed. CNVs (based on molecule coverage) were called against human reference genome (GRCh38/hg38). Data were analyzed with Bionano Access software v.1.7.1 and Bionano Tools on Saphyr Compute Servers (Bionano Genomics).

### 2.5. Long-Range Polymerase Chain Reaction (PCR)

Based on the known upstream and downstream sequences of the two breakpoint regions revealed by OGM, a series of forward and reverse PCR primers was designed (Table S1). Long-range PCR was first executed using primers and LongAmp Taq PCR Kit (E5200S; NEB, Ipswich, MA). PCR conditions were 96°C for 5 min followed by 35 cycles of 96°C for 30 sec, 60°C for 30 sec, and 72°C for 4 min, with a final extension at 72°C for 4 min.

### 2.6. Targeted Next-Generation Sequencing

Long-range PCR product was subjected to next-generation sequencing. DNA was enriched with the xGen Exome Research Panel v2.0 (Integrated DNA Technologies, Coralville, IA), sequencing on HiSeq 2000/2500 to generated 150 bp paired-end reads (Illumina). Total sequencing depth was 100x. Reads were mapped to reference sequence GRCh38/hg38. Coverage of the region-of-interest was >20. Mapping rate was >96. Reference sequences for *JAG1* and *FAM13A* were NM_000214.3 and NM_014883.4, respectively. ANNOVAR [8] and Ensembl variant effect predictor (VEP) were used for the annotation of SNVs and InDels. Lumpy (v0.2.13) [9] and Delly (v0.8.3) [10] were used for SV calling.

### 2.7. Retrospective SV Calling Using Short-Read Whole Genome Sequencing (S-WGS) Data

Manta v1.6.0 [11] was used for retrospective SV calling as described. Integrative Genomics Viewer (IGV) browser (https://www.igv.org) was used to visualize and accurately pinpoint the breakpoint.

### 2.8. Cytogenetic Analysis

Peripheral blood samples were processed for karyotyping by standard methods. Parental karyotypes (550-band resolution) were obtained in December 2021, at Hefei Anweikang Medical Laboratory (Hefei, China). A patient karyotype (400-band resolution) was obtained in February 2023, at Wanbei Coal and Electricity Group General Hospital (Suzhou, China). Karyotyping was performed using 20 metaphase cells per person. Karyotypes were annotated according to the International System for Human Cytogenetic Nomenclature 2020.

### 2.9. Ethics

This study was approved by the Ethics Committee of Children's Hospital, Fudan University (No. 2020-402). A parent of the patient signed the written informed consent.

## 3. Results

**Figure 1:**
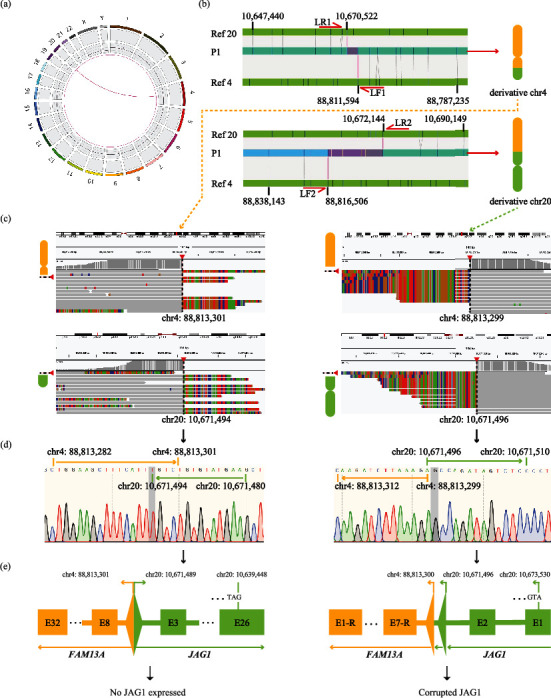
Reciprocal translocation disrupting *JAG1* identified in P1. (a) Circos plot demonstrated a translocation between chromosomes 4 and 20. The Circos plot is composed of four circles (*viz*., four tracks). The outmost track with number (1,2,3…X, Y) stands for simulated cytoband on each chromosome; the second track with colorful dots represents SVs (including InDel, inversion, and duplication); the third track with a central purple line exhibits CNVs (purple marks a baseline; outward blue and inward red indicate CNV gain and CNV loss segments, individually); the inmost track with purple circle and crossed lines shows translocation (intra- and intermolecules). (b) Reciprocal translocation presented in genome browser view of OGM. The top and bottom two grass green bars represent reference chr20 and chr4, respectively. The ice green bar in the middle represents the patient's derivative chromosome as matched to reference chr4 and chr20 (gray lines between grass green bar and ice green bar show alignment; purple line suggests breakpoint junction). The number on the reference chromosome corresponding to the two sides of the breakpoint junction represents the position on the chromosome. Ref: GRCh38/hg38 reference sequence of chromosome; LF and LR were primer pairs designed for long-range (L) PCR; chr: chromosome. Schematic derivative chromosome rightmost (chr4 in orange and chr20 in green). (c) Visualization of breakpoints identified by targeted next-generation sequencing. Arrow with dotted line indicates breakpoint. Breakpoints for derivative chr4, left, and for chr20, right. (d) Results of the Sanger sequencing confirming breakpoints for derivative chr4 and chr20. (e) Schematic of *JAG1* and *FAM13A* rearrangement and corresponding damage to JAG1. E: exon; E-R: reverse strand of exon.

In this boy with clinically suspected ALGS, no causative variant in *JAG1* or *NOTCH2* was revealed by panel sequencing and S-WGS. No CNVs in *JAG1* were observed by MLPA analysis (Figure S2). OGM indicated the heterozygous balanced translocation t(4;20)(q22.1;p12.2) (Figure 1 (a) and suggested chr20:10670522~10672144 and chr4:88811594~88816506 (Figure 1 (b)) as approximate breakpoint ranges. Long-range PCR products with sizes of ~3 kb on derivative chromosome 4 and ~5 kb on derivative chromosome 20 represent the translocated and rearranged segments (Figure S3a and Table S1). Breakpoints and rejoining localizations were visualized (IGV) on chromosome 4 as chr4:88,813,301 rejoined with chr20:10,671,494 (Figure 1 (c)) and on chromosome 20 as chr4:88,813,299 rejoined with chr20:10,671,496 (Figure 1 (c)). Sequencing further confirmed these breakpoints (Figure 1 (d)). This heterozygous translocation leads to a fusion of *JAG1* (exon1-exon2) with *FAM13A* (exon7-exon1) on rearranged chromosome 20, predicted to interrupt *JAG1* transcription and to impair protein expression (Figure 1 (e) and Figure S3c) or to yield nonsense-mediated mRNA decay. *JAG1*, as fused with *FAM13A* on derivative chromosome 4, could not be transcribed and translated as start codon ATG is absent (Figure 1 (e), left).

**Figure 2:**
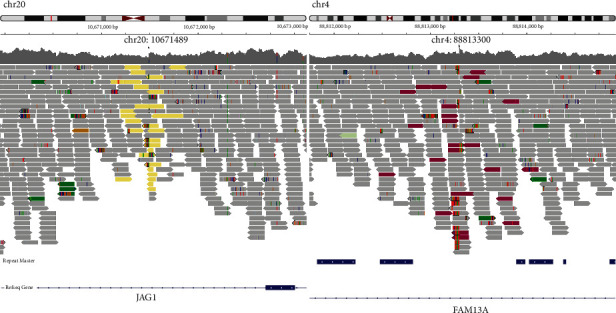
Breakpoints indicated in whole genome sequencing (WGS) of the patient. Reinvestigating the WGS data for the patient identified compatible breakpoints on chromosome 4 and 20: chr20:10671489 and chr4:88813300 (black arrows). The majority of sequences matching the reference sequences (GRCh38/hg38) are shown in gray. The yellow bars on the left represent gene sequences mapped to chromosome 4 and abnormally inserted into chromosome 20, while the purple-red bars on the right represent gene sequences mapped to chromosome 20 and abnormally inserted into chromosome 4.

**Figure 3:**
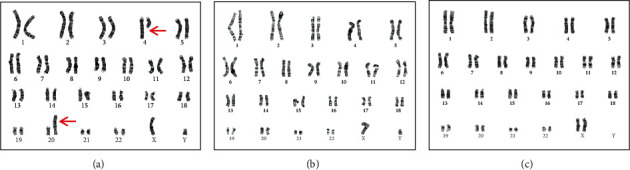
Karyotypes of the patient and his parents. (a) Karyotype analysis (400 bands, G-bands) of the patient shows a balanced translocation (red arrows), with a karyotype of 46,XY,t (4;20)(q22;p12). (b) Karyotype analysis (550 bands, G-bands) of the father shows a normal karyotype of 46,XY. (c) Karyotype analysis (550 bands, G-bands) of the mother shows a normal karyotype of 46,XX.

Retrospective SV calling using S-WGS data found breakpoints on chr4 at 88,813,300 and that on chr20 at 10,671,489 (Figure 2), in accordance with the findings on OGM. Further karyotype analysis confirmed the balanced translocation t(4;20)(q22;p12) (Figure 3(a)). PCR and karyotype analysis showed that this translocation was absent in his healthy parents, indicating *de novo* origin (Figure S3a and S3b and Figures 3(b) and 3(c)). The patient was thus genetically diagnosed as ALGS due to *JAG1* variation.

## 4. Discussion

SVs are not unusual in ALGS [6, 12], including large deletions, inversions, and translocations. These are easily missed on sequencing (panel, whole exome, or whole genome). Karyotype analysis and fluorescence *in situ* hybridization (FISH) are traditional methods of chromosome translocation detection, but these offer only low resolution at 10 Mb~100 kb, precluding characterization of mutations at single-gene level. So far, only 5 families affected by ALGS with translocation found by FISH/karyotype analysis are reported [2, 13–16] but without accurate breakpoints defined at base-pair resolution. Long-read whole genome sequencing might address this, but it is not affordable for routine clinical use. Though multiomics may help [17], suitable RNA samples are not always available. More powerful methods of DNA-level detection thus are needed.

OGM is a karyotype-mimic cytogenetic method that detects SVs utilizing ultra-long, megabase-size linear DNA molecules and fluorescence labels at CTTAAG motifs to facilitate *de novo* genome assembly. As a good complement to short-read sequencing, it can visualize DNA structure and reveal a wide spectrum of SVs with robust high efficiency [18–22]. Sequencing is still necessary for breakpoint verification, however, because the images obtained by OGM can only provide size/location flanking around the disrupted region.

This seemingly obvious translocation was missed by S-WGS on original analysis because a routine variant calling pipeline used in the laboratory where that analysis was conducted did not utilize the Manta algorithm for SVs. SV calling using that algorithm was performed retrospectively after OGM identified the translocation. This reanalysis recognized the same translocation as did OGM, with exactly the same breakpoints. Of note is that the Manta algorithm, when deployed for SV calling with no standard guideline and/or without a reference to filter false or benign SVs, usually calls out several hundred translocations per sample.

In this study, OGM in a patient with clinical strongly suspected ALGS revealed that *JAG1* was disrupted by the fusion of *JAG1* on chromosome 20 with *FAM13A* on chromosome 4. Review of all ALGS cases reported in the PubMed database and all variants of *JAG1* included in the Human Gene Mutation Database (HGMD) identified no instance of the same breakpoint in *JAG1*. Our patient thus is the first described ALGS patient who carries a balanced translocation of chromosome 4 and chromosome 20 that interrupts JAG1; one can infer that the breakpoint is not likely a spot at which disruption recurs. DNA strand breaks may be related to microhomology sequences or to specific recognition sequences upstream and downstream of the breakpoint [23, 24]. In this context of interest is that 5 identical bases lie at the breakpoints of chromosome 20 and chromosome 4, *viz.*, the base sequence of chr4:88813297-88813301 is the same as that on the antisense strand of chr20:10671490-10671494 (Figure 1 (d), left).

OGM is powerful in detecting translocation, inversion, tandem repeats, and complex genomic rearrangement. It can also identify chromosomal aneuploidy and microdeletions/microduplications. Clinically, OGM is of particular value in patients with negative results in sequencing. However, at present, OGM can not recognize Robertsonian translocation. Thus for patients highly suspected of harboring Robertsonian translocation, karyotyping is necessary.

In conclusion, this is the first balanced reciprocal translocation characterized at base-pair resolution in ALGS detected by OGM, expanding the pathogenic variation spectrum. This technical approach permitted precise diagnosis and genetic counseling. When routine testing finds no variants in genes strongly implicated by clinical assessment, one should consider using multiple SV calling tools in analysis of next-generation sequencing data and, in addition or as an alternative, OGM.

## Figures and Tables

**Table 1:** Biomarkers for cholestasis and injury of renal tubules at aged 72 days.

Index	Assessment	Reference value
Total bilirubin (*μ*mol/L)	79.2	3.4~17.1
Direct bilirubin (*μ*mol/L)	66.2	0~6
Alanine aminotransferase (IU/L)	273	9~50
Aspartate aminotransferase (IU/L)	220	15~40
Total bile acids (*μ*mol/L)	91	0~10
Gamma-glutamyl transferase (IU/L)	1536	8~57
Total cholesterol (mmol/L)	7.29	0~5.18
*α*1 microglobulin/creatinine (mg/g)	273.8	0~14
Microalbumin/creatinine (mg/g)	60.3	0~26.5
Immunoglobulin/creatinine (mg/g)	40.4	0~14
N-Acetyl-*β*-D-glucosidase/creatinine (U/mmol)	14.44	0.3~1.2

## Data Availability

All data relevant to the study are included in the article or uploaded as supplementary information. We have submitted our variation to ClinVar with accession number SCV002583543.

## Additional Points

*Web Resources*. The following are the web resources: ANNOVAR: https://annovar.openbioinformatics.org/en/latest/, BWA: http://bio-bwa.sourceforge.net/, ClinVar: https://www.ncbi.nlm.nih.gov/clinvar/, Delly: https://github.com/dellytools/delly, ExAC: http://exac.broadinstitute.org, FastQC: https://www.bioinformatics.babraham.ac.uk/projects/fastqc/, GATK: https://software.broadinstitute.org/gatk/best-practices/, gnomAD: https://gnomad.broadinstitute.org/, HGMD: http://www.hgmd.cf.ac.uk/ac/index.php, IGV: http://software.broadinstitute.org/software/igv/, Lumpy: https://github.com/arq5x/lumpy-sv, Manta: https://github.com/Illumina/manta, OMIM: https://www.ncbi.nlm.nih.gov/omim, SpeedSeq: https://github.com/hall-lab/speedseq, UCSC: http://genome.ucsc.edu, VEP: http://asia.ensembl.org/info/docs/tools/vep/index.html, and 1000G: https://www.internationalgenome.org/.

## Supplementary Materials

Supplementary 1 Table S1 lists primers used in breakpoint identification and confirmation.

Supplementary 2 Figure S1 shows an anteroposterior radiograph of the spine.

Supplementary 3 Figure S2 shows the results of MLPA analysis of JAG1.

Supplementary 4 Figure S3 shows agarose electrophoretogram of PCR products and predicted sequence on both DNA and amino-acid levels.
