# Supplementary material for: Balanced Translocation Disrupting JAG1 Identified by Optical Genomic Mapping in Suspected Alagille Syndrome
Source: Hum Mutat. 2023 Jun 8;2023:5396281. doi: 10.1155/2023/5396281 (PMC11918711; doi:10.1155/2023/5396281)
Supplement: Supplementary 1 — Table S1 lists primers used in breakpoint identification and confirmation. [file 5396281.f1.docx]

**Supporting information**

**Balanced translocation disrupting *JAG1* identified by optical genomic mapping in suspected Alagille syndrome**

**Authors**: Yi-Qiong Zhang^1^**^#^**, Peng-Fei Gao^2#^, Jing-Min Yang^2,3,4^, Jing Zhang^1^, Yu-Lan Lu^5^, Jian-She Wang ^1,6*^

**Supplementary data**

| **Table S1. Primers for confirmation of the breakpoint junctions on chromosome 4 and 20** | | | | |
| --- | --- | --- | --- | --- |
| Test method | Primers (5’-3’) | Location of primers | Targeted chromosome | Length of PCR product |
| Long-range PCR | LF1: TGGCATTCTTTATCACCTATTCTGTC  LR1: TTGGGAGGACTCATGCAAATGGTAA | chr4(+):88811391-88811416  chr20(+):10670300-10670324 | derivative chr4 | ~3kb |
|  | LF2: CTTTTGTGAGTAGTTTCTTCCCCTTGGT  LR2:TCGGGTCAGTTCGAGTTGGAGATCCTGT | chr4(-):88817106-88817133  chr20(-):10672967-10672994 | derivative chr20 | ~5kb |
| PCR | SF1: CCATGGGTTTTCTCCCATCCTT  SR1: GGCCAGCCAGATTTAAACGC | chr4(+): 88813002-88813023  chr20(+):10671204-10671223 | derivative chr4 | 586bp |
|  | SF2: ACAGGTATCCCCATATTGTTTTGA  SR2: GGGTGGAAGGAAGATGGGTG | chr4(-):8813490-88813513  chr20(-):10671752-10671771 | derivative chr20 | 490bp |
| Location on chromosome was based on reference GRCh38/hg38. kb, kilo-base pair. bp, base pair.  +, the sense strand oriented in the 5’-3’ direction; -, the complementary strand oriented in the 3’-5’ direction. | | | | |
