# Supplementary figures and images for: Balanced Translocation Disrupting JAG1 Identified by Optical Genomic Mapping in Suspected Alagille Syndrome
Source: Hum Mutat. 2023 Jun 8;2023:5396281. doi: 10.1155/2023/5396281 (PMC11918711; doi:10.1155/2023/5396281)

Figure S1

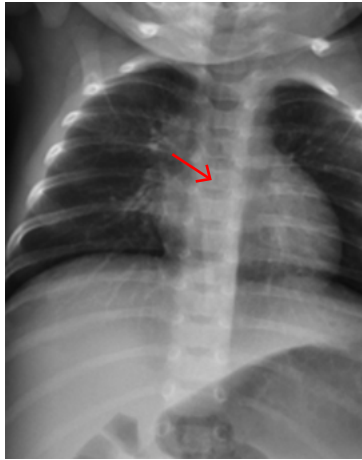

Figure S1. Anteroposterior radiograph of the spine  
Note vertebral cleft (red arrow).

Supplement: Supplementary 2 — Figure S1 shows an anteroposterior radiograph of the spine. [file 5396281.f2.pdf]
