# Supplementary material for: Balanced Translocation Disrupting JAG1 Identified by Optical Genomic Mapping in Suspected Alagille Syndrome
Source: Hum Mutat. 2023 Jun 8;2023:5396281. doi: 10.1155/2023/5396281 (PMC11918711; doi:10.1155/2023/5396281)
Supplement: Supplementary 3 — Figure S2 shows the results of MLPA analysis of JAG1. [file 5396281.f3.pdf]

Figure S2

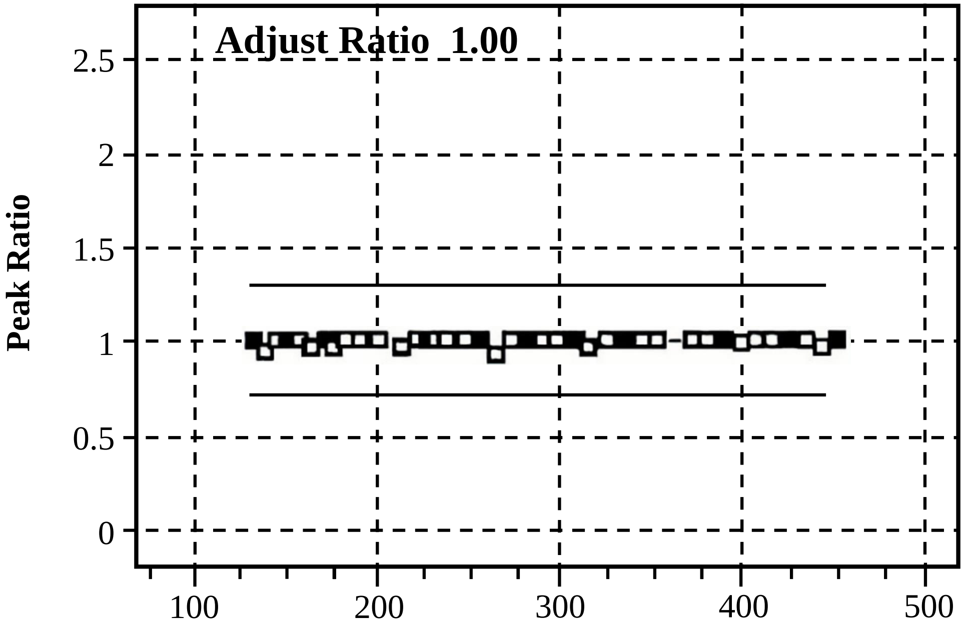

Figure S2. Results of multiplex ligation-dependent probe amplification (MLPA) analysis for *JAG1*.
MLPA analysis identified no copy number variants in *JAG1*.
