# Supplementary material for: Balanced Translocation Disrupting JAG1 Identified by Optical Genomic Mapping in Suspected Alagille Syndrome
Source: Hum Mutat. 2023 Jun 8;2023:5396281. doi: 10.1155/2023/5396281 (PMC11918711; doi:10.1155/2023/5396281)
Supplement: Supplementary 4 — Figure S3 shows agarose electrophoretogram of PCR products and predicted sequence on both DNA and amino-acid levels. [file 5396281.f4.pdf]

Figure S3

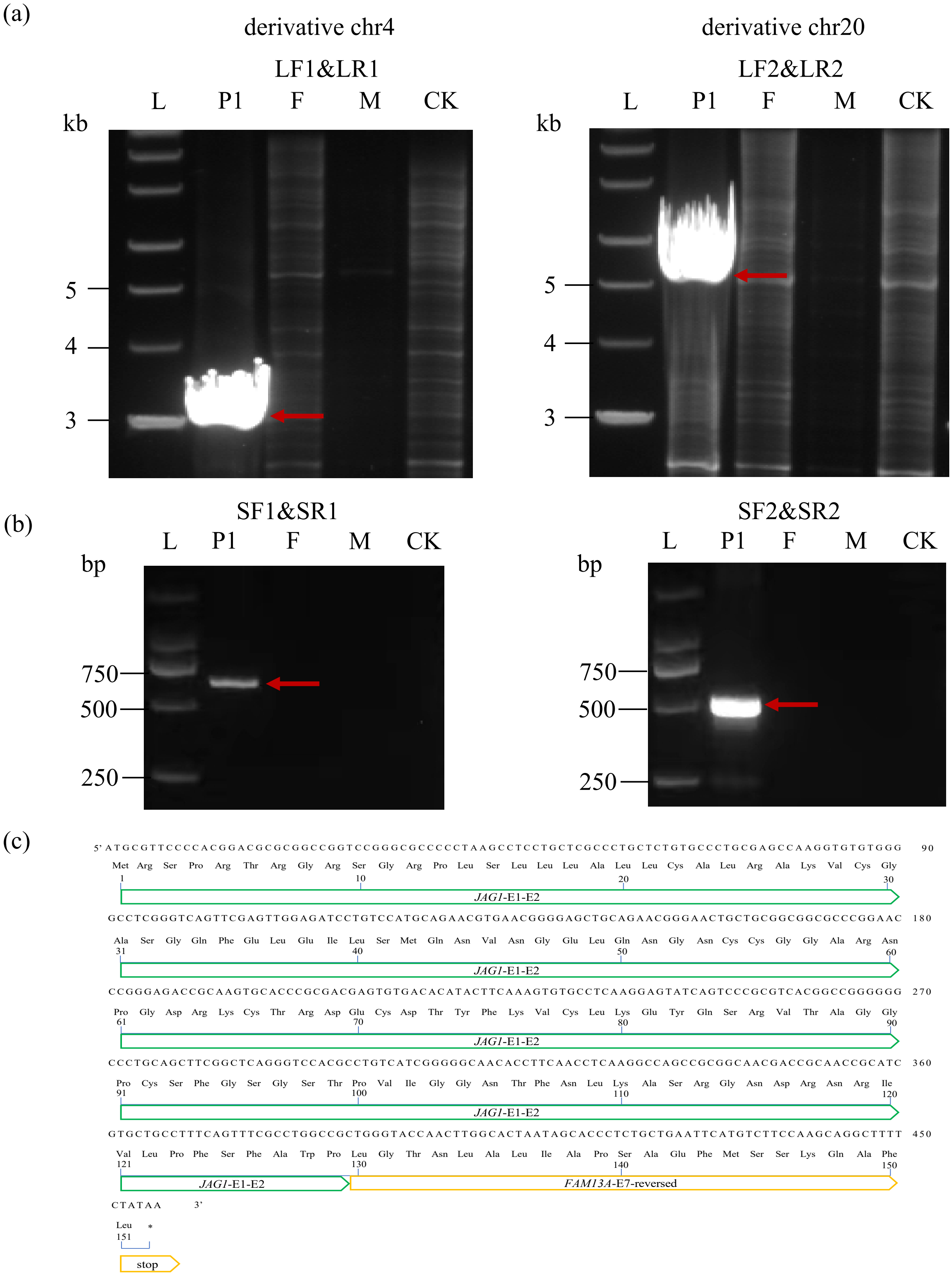

(a)
derivative chr4
LF1&LR1
L P1 F M CK
kb
5
4
3
derivative chr20
LF2&LR2
L P1 F M CK
kb
5
4
3
(b)
SF1&SR1
L P1 F M CK
bp
750
500
250
SF2&SR2
L P1 F M CK
bp
750
500
250
(c)
5' ATGCGTTCCCCACGGACGCGCGGCCGGTCCGGGCGCCCCCTAAGCCTCCTGCTCGCCCTGCTCTGTGCCCTGCGAGCCAAGGTGTGTGGG 90
Met Arg Ser Pro Arg Thr Arg Gly Arg Ser Gly Arg Pro Leu Ser Leu Leu Leu Ala Leu Leu Cys Ala Leu Arg Ala Lys Val Cys Gly
1 10 20 30
JAG1-E1-E2
GCCTCGGGTCAGTTCGAGTTGGAGATCCTGTCCATGCAGAACGTGAACGGGGAGCTGCAGAACGGGAACTGCTGCGGCGGCGCCCGGAAC 180
Ala Ser Gly Gln Phe Glu Leu Glu Ile Leu Ser Met Gln Asn Val Asn Gly Glu Leu Gln Asn Gly Asn Cys Cys Gly Gly Ala Arg Asn
31 40 50 60
JAG1-E1-E2
CCGGGAGACCGCAAGTGCACCCGCGACGAGTGTGACACATACTTCAAAGTGTGCCTCAAGGAGTATCAGTCCCGCGTCACGGCCGGGGGG 270
Pro Gly Asp Arg Lys Cys Thr Arg Asp Glu Cys Asp Thr Tyr Phe Lys Val Cys Leu Lys Glu Tyr Gln Ser Arg Val Thr Ala Gly Gly
61 70 80 90
JAG1-E1-E2
CCCTGCAGCTTCGGCTCAGGGTCCACGCCTGTCATCGGGGGCAACACCTTCAACCTCAAGGCCAGCCGCGGCAACGACCGCAACCGCATC 360
Pro Cys Ser Phe Gly Ser Gly Ser Thr Pro Val Ile Gly Gly Asn Thr Phe Asn Leu Lys Ala Ser Arg Gly Asn Asp Arg Asn Arg Ile
91 100 110 120
JAG1-E1-E2
GTGCTGCCTTTCAGTTTCGCCTGGCCGCTGGGTACCAACTTGGCACTAATAGCACCCTCTGCTGAATTCATGTCTTCCAAGCAGGCTTTT 450
Val Leu Pro Phe Ser Phe Ala Trp Pro Leu Gly Thr Asn Leu Ala Leu Ile Ala Pro Ser Ala Glu Phe Met Ser Ser Lys Gln Ala Phe
121 130 140 150
JAG1-E1-E2
FAM13A-E7-reversed
CTATAA 3'
Leu *
151
stop

Figure S3. Results of agarose electrophoresis for PCR products and prediction of abnormal JAG1.
(a) PCR products amplified by long-range PCR using primer pairs LF1/LR1 and LF2/LR2 for derivative chromosome 4 and 20 individually (1% agarose). Bands indicated with red arrows were subjected to sequencing. P1, patient; F, father; M, mother; CK, healthy control; L, DNA ladder; kb, kilobase; bp, basepair.
(b) PCR products amplified by short range PCR using primer pairs SF1/SR1 and SF2/SR2 for derivative chromosome 4 and 20 individually (3% agarose).
(c) *JAG1* exon 1 (E1) and exon 2 (E2), predicted to fuse with *FAM13A* exon 7 (reverse), are expected to generate aberrant *JAG1* transcripts that lead to complete loss of *JAG1* expression.
